# Three-component Castagnoli-Cushman reaction with ammonium acetate delivers 2-unsubstituted isoquinol-1-ones as potent inhibitors of poly(ADP-ribose) polymerase (PARP)

**DOI:** 10.1080/14756366.2021.1969386

**Published:** 2021-08-31

**Authors:** Alexander Safrygin, Petr Zhmurov, Dmitry Dar’in, Sergey Silonov, Mariia Kasatkina, Yulia Zonis, Maxim Gureev, Mikhail Krasavin

**Affiliations:** a Chemistry, Saint Petersburg State University, Saint Petersburg, Russia; b JSC BIOCAD, Saint Petersburg, Russia; c Digital Biodesign and Personalized Healthcare Research Center, Sechenov First Moscow State Medical University, Moscow, Russia

**Keywords:** Castagnoli-Cushman reaction, 1-oxo-3,4-dihydroisoquinoline-4-carboxamides, poly(ADP-ribose) polymerase, PARP1/2 selectivity, NAD^+^ mimetics, druglikeness

## Abstract

An earlier described three-component variant of the Castagnoli-Cushman reaction employing homophthalic anhydrides, carbonyl compound and ammonium acetate was applied towards the preparation of 1-oxo-3,4-dihydroisoquinoline-4-carboxamides with variable substituent in position 3. These compounds displayed inhibitory activity towards poly(ADP-ribose) polymerase (PARP), a clinically validated cancer target. The most potent compound (PARP1/2 IC_50_ = 22/4.0 nM) displayed the highest selectivity towards PARP2 in the series (selectivity index = 5.5), more advantageous ADME prameters compared to the clinically used PARP inhibitor Olaparib.

## Graphical Abstract

**Figure:**
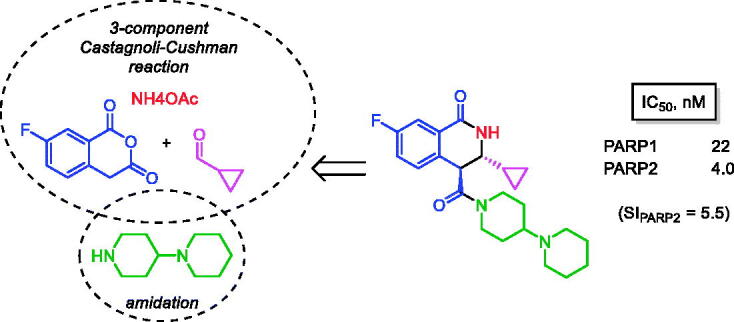

## Introduction

Poly(ADP-ribose) polymerase (PARP) enzymes are regarded as important targets for the development of anticancer drugs owing to the clinical success of PARP inhibitors *Olaparib*, *Talazoparib*, *Niraparib* and *Rucaparib* approved for cancer treatment^1^. PARP1 and PARP2 are two key enzymes that are critical for repairing single-strand breaks (“nicks”) in the DNA – a mechanism which is critical for the survival of both normal and cancer cells^2^. It is generally accepted that, despite the non-selectivity of the approved PARP inhibitors, it is the inhibition of PARP1 that is responsible for the manifestation of their clinical efficacy^3^. Normal cells do not divide as frequently as cancer cells, which allows them to survive PARP1 inhibition. However, tumour cells with certain mutations that are synthetically lethal with PARP1 inhibition^4^ are efficiently killed by the drugs of this class. This notion motivated the development of selective PARP1 inhibitors such as NMS-P118 reported by Nerviano Medical Sciences^5^.

**Figure 1.**
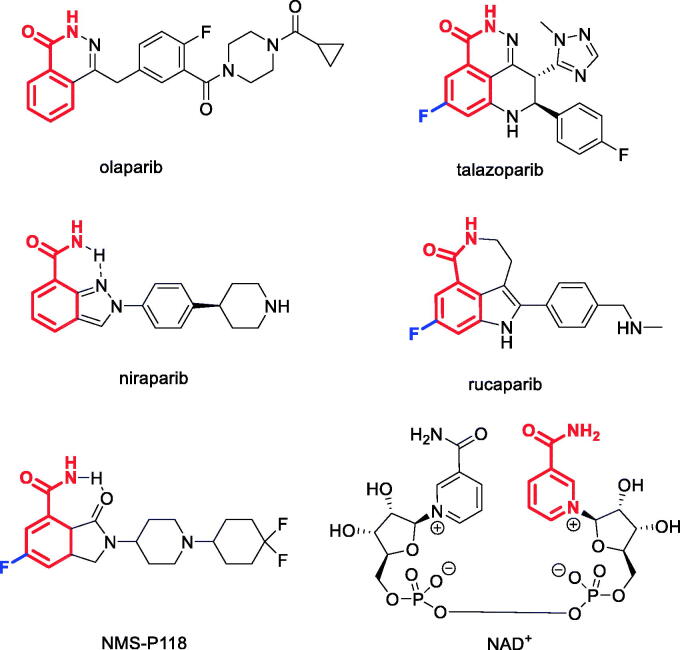
Clinically used PARP1 inhibitors, advanced clinical candidate NMS-P118 (NAD+-mimicking motif is highlighted in red, NAD^+^ structure shown).

The majority of PARP1 inhibitors, including the above-mentioned “*paribs*”, were designed to mimic the nicotinamide moiety of NAD^+^ (from which the adenine ribose unit of poly(ADP-ribose) originates) with which the inhibitors compete for the NAD^+^-binding site of PARP1. This mimicry is achieved *via* the use of either a rotationally constrained primary benzamide (as in Niraparib and NMS-P118) or a benzamide motif embedded in a ring (as in Olaparib, Talazoparib and Rucaparib). Another characteristic feature noticeable in some of the advanced PARP1 inhibitors is the presence of a fluorine atom (the “magic fluorine” highlighted in blue) in the *meta*-position of the NAD^+^-mimicking benzamide moiety (Figure 1). This “magic fluorine” has been shown to enhance binding to the target^5^.

**Figure 2.**
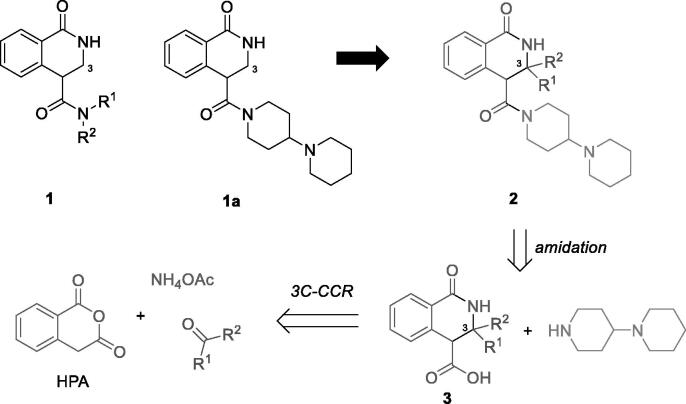
Previously discovered series of PARP1/2 inhibitors **1** with the lead compound **1a**, the newly designed series of inhibitors **2** bearing substitutions at position 3 and its envisioned synthesis *via* the 3 C-CCR.

Recently, we discovered 1-oxo-3,4-dihydroisoquinoline-4-carboxamides **1** as a novel chemotype capable of delivering potent PARP inhibitors. After screening the amide residues around the basic core, we identified compound **1a** as a lead structure for further development. It displayed a potent inhibition profile towards PARP1 (IC_50_ = 156 nM) and PARP2 (IC_50_ = 70.1 nM). Moreover, compound **1a** displayed a better microsomal and plasma stability *in vitro* compared to clinically used PARP1 inhibitor Olaparib^6^. Encouraged by this finding, we continued looking for ways to improve the potency profile of the 1-oxo-3,4-dihydroisoquinoline-4-carboxamide series. Carboxamide **1a** bore no substituents in position 3 of the 1-oxo-3,4-dihydroisoquinoline core. Therefore, we considered exploring the structure-activity relationships within the series **2** where the 3-mono- and 3,3-disubstituted scaffolds could be screened while the 1,4′-bipiperidine carboxamide would remain unchanged. Access to derivatives **2** was envisioned via the amidation of carboxylic acids **3** which, in turn, could be prepared *via* the recently reported three-component Castagnoli-Cushman reaction (3 C-CCR) of homophthalic anhydride (HPA), carbonyl compounds and ammonium acetate^7^ (Figure 2). Herein, we describe the results obtained in the course of realising this strategy.

## Results and discussion

**Scheme 1.**
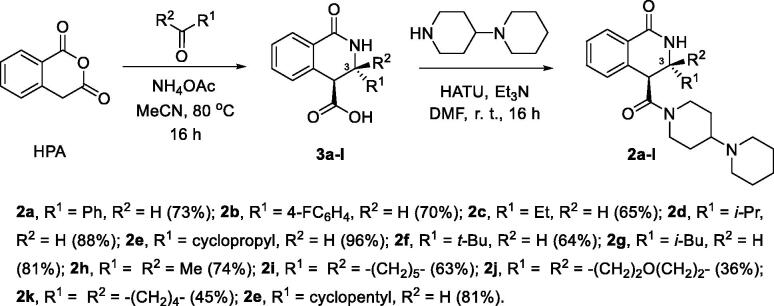
Synthesis of 3-substituted 1,4'-bipiperidine 1-oxo-3,4-dihydroisoquinoline-4-carboxamides **2a-l**.

Twelve carboxylic acids **3a-l** were synthesised in diastereomerically pure (*trans*-configured, based on their vicinal coupling constants of methine protons) form as described previously^7^ and subjected to amidation with 1,4′-bipiperidine using 1-[bis(dimethylamino)methylene]-1*H*-1,2,3-triazolo[4,5-*b*]pyridinium 3-oxide hexafluorophosphate (HATU) to activate the carboxylic group^6^. Resulting amides **2a-l** were obtained in moderate to excellent yields (Scheme 1).

**Scheme 2.**
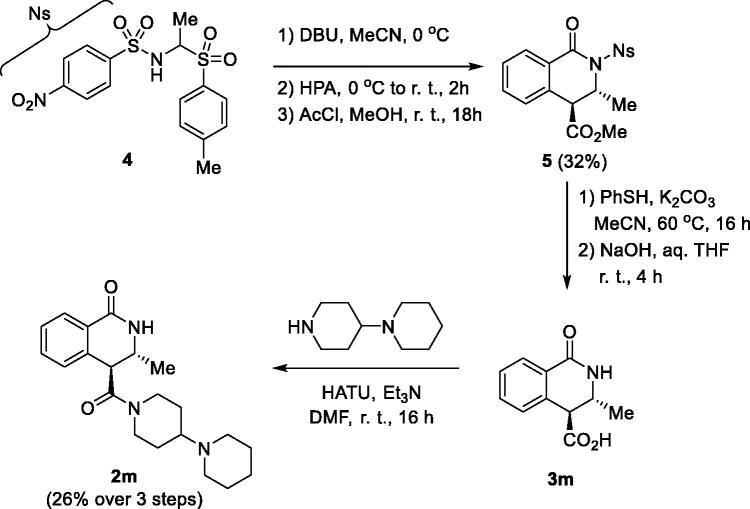
Synthesis of 3-methyl-substituted 1-oxo-3,4-dihydroisoquinoline-4-carboxamide **2 m**.

In order to probe for the minimal substitution at position 3, we aimed to synthesise 3-methyl-substituted 1-oxo-3,4-dihydroisoquinoline-4-carboxylic acid **3 m**. Unfortunately, the 3 C-CCR protocol did not furnish the desired product, likely due to the high volatility of acetaldehyde. Thus, for the preparation of **3 m**, we resorted to the alternative approach recently reported by Shaw and co-workers^8^. It involves *in situ* generation of *N*-(4-nitrobenzene)sulfonyl (4-nosyl) imine of acetaldehyde from α-aminosulfone **4**^9^ on treatment with equimolar amount of base. *N*-Sulfonyl imine 4 thus generated can be reacted with HPA in the Castagnoli-Cushman fashion. Indeed, treatment of **4** with 1 equiv. of DBU followed by the addition of HPA furnished 4-nosyl CCR adduct which was esterified to give ester **5** in modest yield. Removal of the 4-nosyl protecting group followed by ester hydrolysis furnished the target acid (**3 m**) which was immediately introduced in the amidation reaction with 1,4′-bipiperidine to give compound **2 m** in modest 26% yield over 3 steps (Scheme 2).

Compounds **2a-m** (confirmed to retain the trans-configuration based on the values of *^3 ^J*(H^3^-H^4^) coupling constants observed in their ^1^H NMR spectra) were tested for inhibitory activity towards PARP1 and PARP2 using the commercially available colorimetric activity assay kit from BPS Bioscience (San Diego, CA) in full accordance of the supplier’s method description^10–11^. The initial screening was performed against PARP1 at 1 µM concentration of each compound, in triplicate (*n* = 3) measurements. Compounds which displayed over 80% inhibition of the enzyme activity were in dose-response mode (*n* = 3) against PARP1 and PARP2 using Olaparib as the reference inhibitor in order to determine the compounds’ half-maximal inhibitory concentration (IC_50_) and assess the isoform selectivity (Table 1).

**Table 1.** Inhibitory activity of compounds **2a-m** *vs.*

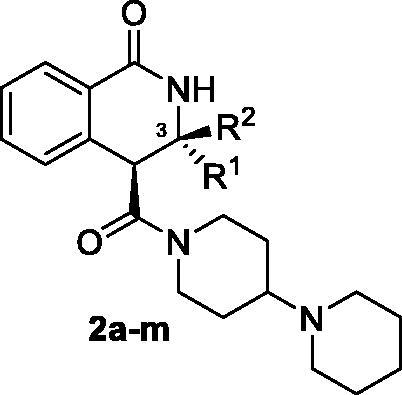					
Compound	R1	R2	IC50, nM (or % inhibition at 1 µM)a		SIb
			PARP1	PARP2	
**2a**	Ph	H	87.9 ± 10.2	69.5 ± 12.8	1.26
**2b**	4-FC_6_H_4_	H	55.4 ± 8.8	39.9 ± 7.3	1.39
**2c**	Et	H	44.6 ± 5.6	11.0 ± 2.2	4.05
**2d**	*i*-Pr	H	159.9 ± 18.6	ND^c^	–
**2e**	Cyclopropyl	H	26.4 ± 4.1	18.3 ± 4.5	1.44
**2f**	*t*-Bu	H	24.1 ± 6.0^d^	–	–
**2g**	*i*-Bu	H	87.0 ± 8.3	104.9 ± 20.8	0.83
**2h**	Me	Me	4.0 ± 7.4^d^	–	–
**2i**	-(CH_2_)_5_-		12.1 ± 7.2^d^	–	–
**2j**	-(CH_2_)_2_O(CH_2_)_2_-		5.1 ± 6.6^d^	–	–
**2k**	-(CH_2_)_4_-		10.6 ± 9.7^d^	–	–
**2l**	Cyclopentyl	H	>500	>500	–
**2m**	Me	H	95.7 ± 17.7	29.2 ± 3.4	3.28
**1a** ^e^	H	H	156 ± 5.8	70.1 ± 7.6	2.23
Olaparib			2.8 ± 0.3	0.7 ± 0.2	3.98

^a^The data are presented as the mean value ± SD obtained in triplicate (*n* = 3) measurement of the enzyme activity.

^b^Selectivity index defined as IC_50_(PARP1)/IC_50_(PARP2), i.e. values greater than 1.0 indicate inhibitor’s selectivity towards PARP2.

^c^Not determined.

^d^The value = % inhibition at 1 µM concentration of the inhibitor.

^e^Data from [6].

PARP1 and PARP2 enzymes.

It is apparent from the data presented in Table 1 that some small alkyl groups introduced in position 3 of the 1-oxo-3,4-dihydroisoquinoline-4–1,4′-bipiperidine carboxamide scaffold increase compounds’ potency towards PARP1 and PARP2 (*cf.* **2c**, **2e** (the most potent inhibitor in the series) and **2 m** *vs.* **1a**) while bulkier (cyclo)alkyl groups do not change (**2d**) or ablate (**2f** and **2 l**) the inhibitor’s potency. Similarly, disubstituted (**2 h**) and spirocyclic (**2i-k**) analogs do not inhibit PARP1 in the concentration range relevant for drug development^12^. In light of the bulky substituents at position 3 being detrimental to the inhibitor’s activity, it came as a surprise that 3-aryl-substituted analogs **2a-b** displayed better potency compared to their unsubstituted counterpart (**1a**).

**Figure 3.**
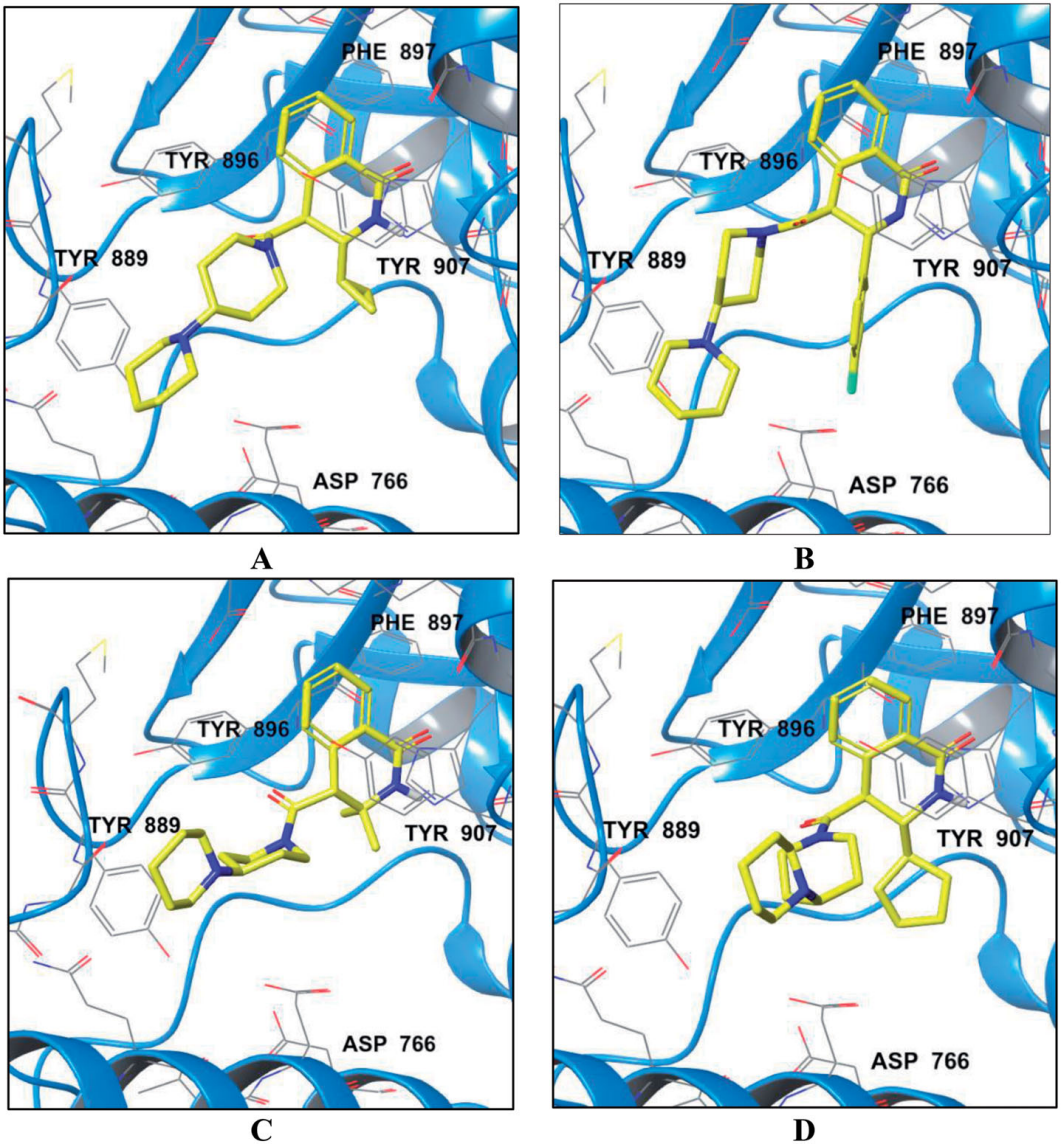
Binding poses of compounds **2e** (A), **2 b** (B), **2 h** (C) and **2 l** (D) in the active site of PARP1; crystal structure of PARP1 with inhibitor NMS-P118 (PBD ID 4ZZZ) was used for docking.

We attempted to rationalise some of the observed structure-activity relationships by performing docking simulation of the inhibitors’ binding to the PARP1 active site. Figure 3 displays the binding poses of the most potent compound **2e** as well as its active (**2 b**) and inactive (**2 h** and **2 l**) analogs. Compounds **2e** and **2 b** displayed a favourable network of lipophilic contacts in the PARP-1 active cavity (Figure 2(A,B)). The 3,3-dimethyl substitution in **2 h** causes a transition to another metastable conformation with the loss of hydrophobic interactions with Val762 and destabilisation of electrostatic contacts with Glu763/Asp766 (Figure 2(C)). In the case of **2 l**, the cyclopentyl moiety appears to induce a conformational rearrangement of molecule. The resulting reorientation of the 1,4′-bipiperidine moiety leads to the loss of the hydrophobic contacts with Val762 and π-stacking interaction with Tyr907 (Figure 2(D)).

**Scheme 3.**
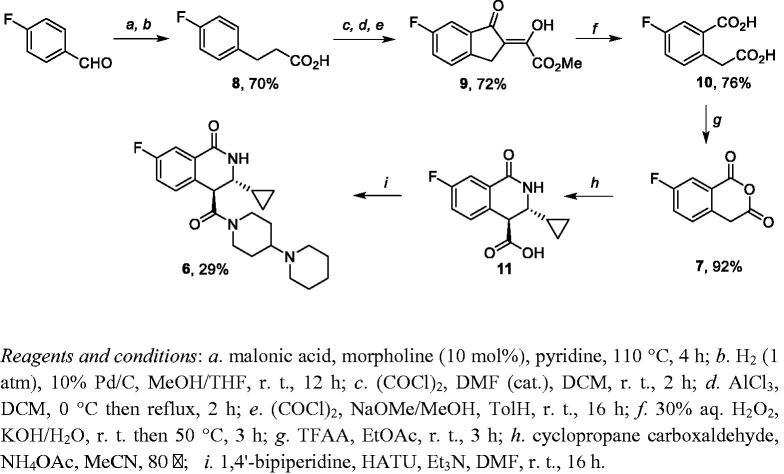
Synthesis of 3-cyclopropyl-substituted 1-oxo-7-fluoro-3,4-dihydroisoquinoline-4-carboxamide **6**. *Reagents and conditions*: *a*. malonic acid, morpholine (10 mol%), pyridine, 110 °C, 4 h; *b*. H_2_ (1 atm), 10% Pd/C, MeOH/THF, r. t., 12 h; *c*. (COCl)_2_, DMF (cat.), DCM, r. t., 2 h; *d*. AlCl_3_, DCM, 0 °C then reflux, 2 h; *e*. (COCl)_2_, NaOMe/MeOH, TolH, r. t., 16 h; *f*. 30% aq. H_2_O_2_, KOH/H_2_O, r. t. then 50 °C, 3 h; *g*. TFAA, EtOAc, r. t., 3 h; *h*. cyclopropane carboxaldehyde, NH_4_OAc, MeCN, 80 °C; *i.* 1,4'-bipiperidine, HATU, Et_3_N, DMF, r. t., 16 h.

Thus, we have identified a new lead compound based on unsubstituted 1-oxo-3,4-dihydroisoquinoline core, **2e**, which inhibited PARP1 with an IC_50_ of 26.4 ± 4.1 nM and PARP2 with an IC_50_ of 18.3 ± 4.5 nM, which corresponded to the selectivity index (SI) of 1.44. Mindful of the advantageous influence of the fluorine atom positioned in the *meta*-position relative to the NAD^+^-mimicking lactam (or carboxamide) moiety, we set off to synthesise a 7-fluoro analog of compound **2e**, 4-([1,4′-bipiperidine]-1′-carbonyl)-3-cyclopropyl-7-fluoro-3,4-dihydroisoquinolin-1(2*H*)-one (**6**). This required that 7-homophthalic anhydride (**7**) be synthesised. Adaptation of its synthesis from the literature^13^ involved 1) the preparation of 3–(4-fluorophenyl)propionic acid (**8**) *via* the Knoevenagel condensation and hydrogenation, 2) indanone ring closure followed by methoxalylation to give compound **9**, 3) oxidative fragmentation of the latter on treatment with hydrogen peroxide and cyclodehydration of 2-(carboxymethyl)-5-fluoro benzoic acid (**10**). The 3 C-CCR involving 7-F-HPA (**7**) followed by amidation (as described above for the preparation of compounds **2a-l**) furnished 7-fluoro analog **6** in modest yield over 2 steps (Scheme 3).

To our delight, when tested for inhibition of PARP1 and PARP2, compound **6** indeed displayed an improved potency profile towards both enzymes (PARP1 IC_50_ = 22.0 ± 3.2 nM, PARP2 IC_50_ = 4.3 ± 0.5 nM, SI = 5.1). Moreover, this compound can be regarded as a relatively rare PARP2-selective inhibitor which can enrich the toolbox of compounds needed for the investigation of the physiological role of PARP2 inhibition^14–16^.

We were curious to compare the physicochemical and ADME properties of the newly discovered lead compound (**6**) to those of more potent, clinically used PARP1/2 inhibitor Olaparib. Indeed, as was noted previously, the sheer potency of pharmacological agents is not a sole determinant of the pharmacodynamic efficacy and should be considered in combination with the overall candidate’s profile^17^.

While, of course, the approved PARP1 inhibitor Olaparib is well within the limits of druglikeness as defined by Lipinsky^18^ (and so is compound **6**), the two compounds are quite similar in terms of molecular weight and lipophilicity. Quite reassuringly, compound **6** displayed similar stability in plasma to that of Olaparib. However, the metabolic stability of the former is significantly higher (Table 2).

**Table 2.** Experimentally determined ADME parameters and calculated molecular characteristics for the lead compound **6** as well as clinically used drug Olaparib.

Compound	Solubility^a^	Stability			PPB^e^	Calculated parameters^19^			
		S9^b^	HLM^c^	Plasma^d^		MW	cLogP	HBD	HBA
Olaparib	>100	1.1	15	100	90.2	434.5	2.52	1	7
**6**	>100	0.1	3.8	100	71.0	399.4	2.59	1	5

^a^Solubility (µM) in pH 7.4 0.01 M phosphate buffer solution; ^b^Stability in the presence of hepatic S9 fraction (µL/min/mg); ^c^Stability in the presence of human liver microsomes (µL/min/mg); ^d^% compound remaining after incubation (4 h) with human plasma; ^e^plasma protein binding (% bound).

In summary, we have further optimised the inhibitory potency of an earlier discovered lead **1a** based on a 1-oxo-3,4-dihydroisoquinoline-4-carboxamide scaffold by exploring various substitutions at position 3. This exploration was reliant on the earlier described three-component variant of the Castagnoli-Cushman reaction of ammonium acetate. The profiling of various 3-mono- and 3,3-disubstituted analogs of compound **1a** for PARP1 and PARP2 inhibition allowed establishing structure-activity relationships and led to the identification of 3-cyclopropyl substituent as the preferred periphery pattern. Introduction of a fluorine atom in the position 7 of the 1-oxo-3,4-dihydroisoquinoline core (inspired by the presence of this “magic fluorine” in several clinical candidates and approved drugs) further improved the potency profile and led to 1-oxo-7-fluoro-3,4-dihydroisoquinoline-4-carboxamide **6** as the new lead compound. It displayed 5.5-fold selectivity towards PARP2, good metabolic and plasma stability as well as better plasma protein binding, compared to Olaparib.

## References

[1] Park M-T, Lee S-J. Cell Cycle and Cancer. J Biochem Mol Biol 2003;36:60–5. 12542976 10.5483/bmbrep.2003.36.1.060

[2] Gibson BA, Kraus WL. New insights into the molecular and cellular functions of poly(ADP-ribose) and PARPs. Nat Rev Mol Cell Biol 2012;13:411–24. 22713970 10.1038/nrm3376

[3] Mateo J, Lord CJ, Serra V, et al. A decade of clinical development of PARP inhibitors in perspective. Ann Oncol 2019;30:1437–47. 31218365 10.1093/annonc/mdz192 PMC6771225

[4] Lord CJ, Ashworth A. PARP inhibitors: synthetic lethality in the clinic. Science 2017;355:1152–8. 28302823 10.1126/science.aam7344 PMC6175050

[5] Papeo G, Posteri H, Borghi D, et al. Discovery of 2-[1-(4,4-Difluorocyclohexyl)piperidin-4-yl]-6-fluoro-3-oxo-2,3-dihydro-1H-isoindole-4-carboxamide (NMS-P118): a potent, orally available, and highly selective PARP-1 inhibitor for cancer therapy. J Med Chem 2015;58:6875–98. 26222319 10.1021/acs.jmedchem.5b00680

[6] Safrygin A, Zhmurov P, Dar’in D, et al. 1-Oxo-3,4-dihydroisoquinoline-4-carboxamides as novel druglike inhibitors of poly(ADP-ribose) polymerase (PARP) with favorable ADME characteristics. J Enzyme Inhib Med Chem. submitted. 10.1080/14756366.2021.1972993 PMC8425678 34482781

[7] Safrygin A, Bakulina O, Dar’in D, Krasavin M. Three-component reaction of homophthalic anhydride with carbonyl compounds and ammonium acetate: new developments. Synthesis 2020;52:2190–5.

[8] Laws SW, Moore LC, Di Maso MJ, et al. Diastereoselective base-catalyzed formal [4 + 2] cycloadditions of N-sulfonyl imines and cyclic anhydrides. Org Lett 2017;19:2466–9. 28474515 10.1021/acs.orglett.7b00468 PMC5576723

[9] Hernando E, Arrayas RG, Carretero JC. Catalytic asymmetric Mannich reaction of glycine Schiff bases with α-amido sulfones as precursors of aliphatic imines. Chem Commun 2012;48:9622–4. 10.1039/c2cc35160a 22911099

[10] https://bpsbioscience.com/pub/media/wysiwyg/PARPs/80580_1.pdf(PARP1).

[11] https://bpsbioscience.com/pub/media/wysiwyg/PARPs/80581_1.pdf(PARP2).

[12] Hughes JP, Rees S, Kalindjian SB, Philpott KL. Principles of early drug discovery. Br J Pharmacol 2011;162:1239–49. 21091654 10.1111/j.1476-5381.2010.01127.x PMC3058157

[13] Kang B-R, Wang J, Li H, et al. Synthesis and antitumor activity evaluation of 2-arylisoquinoline-1,3(2H,4H)-diones in vitro and in vivo. Med Chem Res 2014;23:1340–9.

[14] Pellicciari R, Camaioni E, Costantino G, et al. On the way to selective PARP-2 inhibitors. Design, synthesis, and preliminary evaluation of a series of isoquinolinone derivatives. ChemMedChem 2008;3:914–23. 18409175 10.1002/cmdc.200800010

[15] Sunderland PT, Woon ECY, Dhami A, et al. 5-Benzamidoisoquinolin-1-ones and 5-(ω-carboxyalkyl)isoquinolin-1-ones as isoform-selective inhibitors of poly(ADP-ribose) polymerase 2 (PARP-2). J Med Chem 2011;54:2049–59. 21417348 10.1021/jm1010918

[16] Gui B, Gui F, Takai T, et al. Selective targeting of PARP-2 inhibits androgen receptor signaling and prostate cancer growth through disruption of FOXA1 function. Proc Natl Acad Sci U S A 2019;116:14573–82. 31266892 10.1073/pnas.1908547116 PMC6642419

[17] Gleeson MP, Hersey A, Montanari D, Overington J. Probing the links between in vitro potency, ADMET and physicochemical parameters. Nat Rev Drug Discov 2011;10:197–208. 21358739 10.1038/nrd3367 PMC6317702

[18] Lipinski CA, Lombardo F, Dominy BW, Feeney PJ. Experimental and computational approaches to estimate solubility and permeability in drug discovery and development settings. Adv Drug Deliv Rev 2001;46:3–26. 11259830 10.1016/s0169-409x(00)00129-0

[19] Calculated using Molinspiration Chemoinformatics tool (https://www.molinspiration.com/).

